# Glycaemic control in type 2 diabetic patients with chronic kidney disease: the impacts on enzymatic antioxidants and soluble RAGE

**DOI:** 10.7717/peerj.4421

**Published:** 2018-03-30

**Authors:** Foo Nian Wong, Kek Heng Chua, Jin Ai Mary Anne Tan, Chew Ming Wong, Umah Rani Kuppusamy

**Affiliations:** 1 Department of Biomedical Science, Faculty of Medicine, University of Malaya, Kuala Lumpur, Malaysia; 2 Academy of Sciences Malaysia, Kuala Lumpur, Malaysia; 3 Department of Medicine, Faculty of Medicine, University of Malaya, Kuala Lumpur, Malaysia

**Keywords:** Chronic kidney disease, Type 2 diabetes, Glycaemic control, Oxidative stress, Enzymatic antioxidants, Glutathione peroxidase, Superoxide dismutase, Soluble RAGE, Haemoglobin A1c

## Abstract

**Background:**

Chronic kidney disease (CKD) is characterised by long-term kidney damage and renal function decline. Diabetic CKD is the principal subtype of kidney disease in Malaysia and is associated with oxidative stress which plays an important role in development and progression of the disease. Glycaemic control slows down the progression of diabetic complications, including diabetic CKD. However, the implication of glycaemic control on enzymatic antioxidants and soluble RAGE (sRAGE) in CKD patients remains elusive. The aim of this study was to investigate the effect of glycaemic control on the levels or activities of glutathione peroxidase (GPx), superoxide dismutase (SOD) and sRAGE in CKD patients.

**Methods:**

A total of 150 CKD patients and 64 non-CKD patients were enrolled. The type 2 diabetic patients in the recruited study participants were categorised based on their glycaemic control; poor glycaemic control (GC) with haemoglobin A1c (HbA1c) > 7% and good GC with HbA1c ≤ 7%. The levels or activities of GPx, SOD and sRAGE in plasma were measured. These biochemical parameters were analysed using Mann–Whitney *U* test and two-way analysis of variance (ANOVA).

**Results:**

The activities of GPx and SOD as well as plasma level of sRAGE were not significantly different among the CKD patients with varying glycaemic control status. Irrespective of diabetes status and glycaemic control status, CKD patients also exhibited lower plasma SOD activities compared with non-CKD patients. Among the non-CKD patients, SOD activities were significantly higher in diabetic patients with good GC than diabetic patients with poor GC. Two-way ANOVA revealed that both CKD status and glycaemic control had an interaction effect on SOD activities in diabetic subjects with and without CKD. Follow-up analysis showed that SOD activities were significantly higher in non-CKD patients with good GC. There were no overall significant differences in GPx activities among the study participants. Furthermore, plasma sRAGE levels were higher in diabetic patients with CKD than those without CKD, regardless of glycaemic control status. There were no interaction effects between CKD status and glycaemic control status on GPx and sRAGE. Instead, CKD status showed significant main effects on these parameters, indicating significant differences between diabetic subjects with CKD and diabetic subjects without CKD.

**Conclusion:**

Glycaemic control did not quantitatively alter GPx, SOD and sRAGE in diabetic CKD patients. Despite the advantages of good glycaemic control, a well-controlled diabetes in CKD did not modulate the activities of enzymatic antioxidants and sRAGE levels, therefore may not be the primary mechanism to handle oxidative stress.

## Introduction

Chronic kidney disease (CKD) is an irreversible and progressive disease that is characterised by kidney damage and renal function decline. In a clinical setting, the renal function in CKD patients is measured in estimated glomerular filtration rate (eGFR) (Levey et al., 2003). Most CKD cases are caused by type 2 diabetes which is characterised by long-term hyperglycaemia attributed to insulin resistance and insulin insufficiency. Hyperglycaemia increases the risk of many diabetic complications, including diabetic kidney disease (Bash et al., 2008).

CKD is associated with enhanced oxidative stress that is triggered when the antioxidants are unable to counteract the harmful oxidative insults caused by excessive production of reactive oxygen species. The enzymatic antioxidants such as superoxide dismutase (SOD) and glutathione peroxidase (GPx) form a part of the antioxidant defense against oxidative insults. Superoxide anion, a highly reactive ROS, is converted by superoxide dismutase SOD to hydrogen peroxide, which is then reduced to water by catalase and GPx (Fukai & Ushio-Fukai, 2011). The activities of enzymatic antioxidants are generally decreased in diabetic patients with CKD (Wong et al., 2016b) and antioxidant therapy has been shown to lower the risk of albuminuria in diabetic patients (Bolignano et al., 2017).

Receptor for advanced glycation end-products (RAGE) is a type of cell surface receptor of immunoglobulin superfamily that binds to an array of ligands, including advanced glycation end-products (AGEs) (Ramasamy, Yan & Schmidt, 2009). AGEs represent a heterogeneous group of molecules, including pentosidine and Nepsilon-(carboxymethyl)lysine, that are produced as a result of glycation of proteins, lipids or nucleic acids by reducing sugars (Gkogkolou & Böhm, 2012). Soluble form of RAGE (sRAGE) is the most commonly described variant of RAGE which circulates in the blood. Ligand-binding to sRAGE circumvents the deleterious events such as oxidative stress, inflammation and apoptosis arising from the RAGE-ligand binding (Kalea, Schmidt & Hudson, 2009). In fact, sRAGE also shows nephroprotective effects such as improvements in albuminuria and renal function in diabetic nephropathy (Wendt et al., 2003).

Haemoglobin A1c (HbA1c) is commonly used as a tool for monitoring diabetes and hyperglycaemia. HbA1c is a form of glycated haemoglobin which is an indicator of average plasma glucose concentration over three months (The International Expert Committee, 2009). Controlling diabetes and hyperglycaemia has beneficial effects against kidney disease. Long-term intensive glycaemic control has been shown to reduce the risk of kidney failure (Wong et al., 2016a). Besides, tight glycaemic control could also lessen the risks of myocardial infarction, onset and progression of albuminuria in diabetic kidney disease (Ruospo et al., 2017).

Despite the favourable outcomes of well-controlled diabetes, the effect of glycaemic control on enzymatic antioxidants and sRAGE in diabetic CKD patients is hitherto unknown. Therefore, the aim of this study was to investigate the impact of glycaemic control status on the activities or levels of GPx, SOD and sRAGE in CKD patients. The present study sheds light on whether poor glycaemic control is associated with diminished activities of enzymatic antioxidants and increased sRAGE levels in diabetic CKD.

## Materials & Methods

### Recruitment of study participants

The study participants selected in this study were a subset of those recruited earlier as described in a published report (Wong et al., 2016b). In the present study, CKD patients were recruited from University Malaya Medical Centre (UMMC), Kuala Lumpur. The CKD patients whose eGFR have been constantly less than 60 ml/min/1.73 m^2^ for at least six months before recruitment. Kidney transplant recipients and patients with acute kidney injury were not enrolled into this study. A total of 150 CKD patients were categorised into diabetic CKD (D-CKD) patients and non-diabetic CKD (ND-CKD) patients. The D-CKD patients (*n* = 116) were diagnosed with type 2 diabetes while the ND-CKD patients (*n* = 34) were those with hypertension (16), chronic glomerulonephritis (nine), obstructive uropathy (three), analgesic nephropathy (two), polycystic kidney disease (two) and IgA nephropathy (two). The D-CKD patients were divided into subgroups based on their HbA1c levels, namely poor glycaemic control (GC) and good GC. The D-CKD patients with poor GC (*n* = 63) had HbA1c > 7% while the D-CKD patients with good GC (*n* = 53) had HbA1c ≤7% (Hartz et al., 2006).

The non-CKD patients who served as controls in this study were also recruited from UMMC. A total of 64 non-CKD patients whose eGFR have been consistent and higher than 60 ml/min/1.73 m^2^ were selected. These non-CKD patients were classified into diabetic (DM) patients (*n* = 46) who had type 2 diabetes, and non-diabetic (non-DM) patients (*n* = 18) who were afflicted with other chronic diseases such as cardiovascular disease, hypertension, neurodegenerative diseases and osteoporosis. The DM patients were also further divided into patients with poor GC (*n* = 26) and good GC (*n* = 20).

Peripheral venous blood of the study participants was collected in EDTA tubes. Plasma was isolated immediately after blood collection through centrifugation for 10 min at 2,000× g. The plasma specimens were kept at −80 °C prior to the quantitation of GPx, SOD and sRAGE. Extra blood specimens were also collected for routine blood tests required for clinical examination of the patients.

This study was approved by the Medical Ethics Committee UMMC with adherence to the Declaration of Helsinki (Ethics Committee/IRB reference number: 982.17). Both verbal and written informed consent were obtained prior to blood collection.

### Routine blood tests

The blood tests were performed using standard laboratory procedures in the Division of Laboratory Medicine, UMMC. The levels or activities of serum creatinine, alanine transaminase (ALT), aspartate aminotransferase (AST), triglycerides, total cholesterol, high-density lipoprotein (HDL) cholesterol, low-density lipoprotein (LDL) cholesterol and fasting glucose were measured in an ADVIA^®^ 2400 Clinical Chemistry System (Siemens Healthcare Diagnostics, Erlangen, Germany). Sodium lauryl sulfate method was used to measure hemoglobin concentration in a XE-5000 Automated Hematology Analyzer (Sysmex Corporation, Kobe, Japan). Ion-exchange high-performance liquid chromatography assay was used for the measurement of glycated haemoglobin (HbA1c) in VARIANT™ II TURBO Hemoglobin Testing System (Bio-Rad, Hercules, CA, USA). Modified 4-variable Modification of Diet in Renal Disease study equation (Levey et al., 2006) was used to calculate the eGFR of the study participants.

### Assays of enzymatic antioxidants

Both Glutathione Peroxidase Assay Kit and Superoxide Dismutase Assay Kit (Cayman Chemical, Ann Arbor, MI, USA) were used to measure the antioxidant activities of plasma glutathione peroxidase (GPx) and plasma superoxide dismutase (SOD) respectively. The measurements were carried out according to the manufacturer’s protocols. The absorbance was measured in a microtiter-plate ELISA reader, Power Wave X 340 (Bio-Tek Instruments Inc., Winooski, VT, USA).

### Soluble RAGE ELISA assay

Human RAGE Quantikine ELISA Kit (R&D Systems, Minneapolis, MN, USA) was used to measure sRAGE in plasma according to the manufacturer’s protocol. This ELISA kit applies the quantitative sandwich enzyme immunoassay technique to target and bind the extracellular domain of the human sRAGE proteins. The absorbance was measured in a Power Wave X 340 microtiter-plate reader (Bio-Tek Instruments Inc., Winooski, VT, USA).

### Statistical tests

The statistical analyses were performed using the Statistical Package for Social Sciences, version 20 (SPSS Inc., Chicago, IL, USA). The significance threshold was set at *P* < 0.05. The normality of data distribution was examined using Shapiro–Wilk test. For dependent variables of non-normal distribution, Kruskal–Wallis test was performed to examine overall differences in medians between two or more groups of study participants. If the differences in medians were proved to be significant, pairwise comparisons between two participant groups were conducted using Mann–Whitney *U* test. The differences in categorical variables between participant groups were analysed using Chi-squared test.

Two-way analysis of variance (ANOVA) was used to analyse the interaction of CKD status (CKD and non-CKD) and glycaemic control status (poor GC and good GC) on GPx, SOD and sRAGE in diabetic subjects (D-CKD and DM patients). Pairwise comparisons were not conducted following significant main effect of CKD status or glycaemic control status because each of them consists of only two levels. Follow-up analyses were performed if the interaction effect between CKD status and glycaemic control status was significant. Simple main effect test was conducted to evaluate the differences in means of dependent variable between CKD patients and non-CKD patients for each glycaemic control status, as well as between poor GC and good GC for each CKD status. Interaction analysis was performed to evaluate whether the difference in means between CKD patients and non-CKD patients of poor GC were equal to the difference in means between CKD patients and non-CKD patients of good GC. A significant interaction reflects that the differences are not equal to each other. Bonferroni approach was used to set corrected significance threshold (*P*_corr_) to control for Type I error in this analysis.

## Results

### Characteristics of study participants

The baseline characteristics of CKD and non-CKD patients are listed in Table 1. Both D-CKD patients and ND-CKD patients showed significantly higher serum creatinine and lower eGFR as compared with DM patients (*P* < 0.001) and non-DM patient (*P* < 0.001), indicating reduced renal function among the CKD patients. The HbA1c levels and fasting glucose levels were higher in D-CKD patients and DM patients than in the ND-CKD patients (*P* < 0.001) and non-DM patients (*P* < 0.001). The fasting glucose levels were slightly lower in ND-CKD patients than in the non-DM patients (*P* < 0.01). There were less biguanide users (*P* < 0.001) but more insulin users (*P* < 0.001) in D-CKD patients than in the DM patients. The usage of ACEI and ARB in non-DM patient group was less than in DM patient group (*P* < 0.05) and ND-CKD patient group (*P* < 0.05). Furthermore, more DM patients were using statins as compared to non-DM patients (*P* < 0.05).

**Table 1:** Baseline characteristics of CKD and non-CKD patients.

	CKD		Non-CKD	
	D-CKD (*n* = 116)	ND-CKD (*n* = 34)	DM (*n* = 46)	Non-DM (*n* = 18)
Age (years)	66.0 (45.0 to 75.0)	64.0 (41.0 to 74.0)	64.0 (45.0 to 75.0)	64.0 (53.0 to 73.0)
Gender (male (%)/female (%))	78 (67.2)/38 (32.8)	22 (64.7)/12 (35.3)^c1^	24 (52.2)/22 (47.8)	6 (33.3)/12 (66.7)
Ethnic groups (Malay (%)/Chinese (%)/Indian (%))	51 (44.0)/40 (34.5)/25 (21.5)^b2^	14 (41.2)/15 (44.1)/5 (14.7)	8 (17.4)/20 (43.5)/18 (39.1)	6 (33.3)/8 (44.5)/4 (22.2)
Urea (mmol/l)	11.3 (3.9 to 34.0)^b3^	11.1 (3.7 to 27.0)^c3^	4.6 (1.7 to 10.7)	4.5 (2.1 to 9.7)
Serum creatinine (µmol/l)	191.0 (103.0 to 567.0)^b3^	189.5 (86.0 to 481.0)^c3^	70.5 (40.0 to 106.0)	61.5 (43.0 to 92.0)
eGFR (ml/min/1.73 m^2^)	28.6 (4.2 to 53.3)^b3^	31.0 (9.0 to 58.0)^c3^	82.3 (65.5 to 146.6)	91.0 (73.08 to 127.04)
CKD stage 3 (%)/4 (%)/5 (%)	56 (48.3)/49 (42.2)/11 (9.5)	18 (52.9)/12 (35.3)/4 (11.8)	N/A	N/A
ALT (U/l)	23.0 (8.0 to 90.0)	26.5 (6.0 to 90.0)	28.0 (10.0 to 79.0)	28.0 (14.0 to 55.0)
AST(U/l)	22.0 (12.0 to 88.0)	22.0 (7.0 to 80.0)	23.0 (13.0 to 61.0)	25.0 (18.0 to 38.0)
Triglyceride (mmol/l)	1.7 (0.5 to 5.2)^b1^	1.7 (0.8 to 5.5)	1.4 (0.5 to 4.2)	1.3 (0.6 to 3.2)
Total cholesterol (mmol/l)	4.4 (2.3 to 9.9)	4.5 (3.3 to 18.1)	4.5 (3.0 to 6.9)	5.0 (3.2 to 7.1)
HDL-cholesterol (mmol/l)	1.0 (0.1 to 3.2)^b1^	1.2 (0.7 to 2.5)	1.3 (0.9 to 2.3)^c1^	1.4 (1.0 to 2.5)
LDL-cholesterol (mmol/l)	2.3 (1.2 to 7.7)	2.4 (1.4 to 15.8)	2.4 (1.1 to 4.5)	2.8 (1.7 to 5.0)
Haemoglobin (g/l)	118.0 (76 to 165.0)^b1^	122.0 (98.0 to 155.0)^c1^	122.0 (113.0 to 148.0)	135.5 (99.0 to 158.0)
HbA1c (%)	7.4 (3.8 to 13.7)^a^	5.6 (4.4 to 7.0)	7.3 (5.1 to 13.2)^c3^	5.7 (4.7 to 6.5)
Fasting glucose (mmol/l)	7.4 (2.9 to 17.8)^a^	5.1 (3.9 to 6.0)^c2^	7.4 (3.2 to 21.1)^c3^	5.5 (4.6 to 6.7)
Use of medications (%)				
Sulfonylureas	48.7	N/A	46.5	N/A
Biguanides	23.4^b3^	N/A	86.1	N/A
Insulin	50.5^b3^	N/A	18.6	N/A
ACEI	42.3	37.5	48.8^c1^	17.7
ARB	41.4	41.7^c1^	25.6	11.8
Statins	91.9	79.2	88.4^c1^	64.7
GPx (nmol/min/ml)	94.2 (50.1 to 180.0)^b2^	97.6 (50.1 to 154.5)	107.9 (47.5 to 145.2)	110.8 (75.6 to 155.4)
SOD (U/ml)	16.6 (2.3 to 76.1)^b3^	16.6 (8.3 to 37.7)^c2^	28.5 (12.8 to 96.5)	26.0 (15.5 to 87.2)
sRAGE (pg/ml)	1,097.0 (224.8 to 6,438.0)^b3^	870.9 (157.9 to 2,296.0)	564.8 (249.9 to 1,702.0)	667.6 (297.9 to 1,115.0)

**Notes.**

Data are expressed as median (range). Categorical variables are expressed as either numbers or percentages.

D-CKD diabetic CKD patients ND-CKD non-diabetic CKD patients DM diabetic patients without CKD, Non-DM non-diabetic patients without CKD N/A not available

a *P* < 0.001 versus ND-CKD.

b1 *P* < 0.05,

b2 *P* < 0.01,

b3 *P* < 0.001 versus DM.

c1 *P* < 0.05,

c2 *P* < 0.01,

c3 *P* < 0.001 versus Non-DM.

The D-CKD patients showed significantly lower medians of plasma GPx activity (*P* < 0.01) and SOD activity (*P* < 0.001) than DM patients. The ND-CKD patients also exhibited decreased median of SOD activity in comparison to non-DM patients (*P* < 0.01). In addition, the median of plasma sRAGE level was significantly elevated in D-CKD patients than in DM patients (*P* < 0.001).

**Table 2:** Renal markers, enzymatic antioxidants and sRAGE in study participants upon stratification of glycaemic control status.

Parameters	Overall significance of difference in medians		CKD patients			Non-CKD patients		
	*χ*^2^	*P*	D-CKD (PGC) (*n* = 63)	D-CKD (GGC) (*n* = 53)	ND-CKD (*n* = 34)	DM (PGC) (*n* = 26)	DM (GGC) (*n* = 20)	Non-DM (*n* = 18)
Urea (mmol/l)	112.37	<0.001	10.1 (3.9 to 24.9)^b^	12.7 (3.9 to 34.0)^c2^	11.1 (3.7 to 27.0)^d3^	4.5 (2.6 to 10.7)	4.7 (1.7 to 6.5)	4.5 (2.1 to 9.7)
Serum creatinine (µmol/l)	133.77	<0.001	185.0 (103.0 to 363.0)^b^	211.5 (117.0 to 567.0)^c2^	189.5 (86.0 to 481.0)^d3^	69.5 (40.0 to 98.0)	74.5 (42.0 to 106.0)	61.5 (43.0 to 92.0)
eGFR (ml/min/1.73m^2^)	136.03	<0.001	30.9 (10.9 to 49.6)^b^	25.2 (4.2 to 53.3)^c2^	31.0 (9.0 to 58.4)^d3^	86.3 (67.6 to 146.6)	78.9 (65.5 to 132.2)	91.0 (73.1 to 127.0)
GPx (nmol/min/ml)	10.64	Not significant	94.2 (50.1 to 180.0)	94.2 (57.7 to 177.4)	97.6 (50.1 to 154.5)	110.0 (70.5 to 145.2)	103.6 (47.5 to 141.8)	110.8 (75.6 to 155.4)
SOD (U/ml)	54.34	<0.001	17.1 (2.3 to 76.1)^b^	15.9 (8.1 to 64.7)^c2^	16.6 (8.3 to 37.7)^d2^	24.4 (12.8 to 96.5)^c2^	35.7 (22.2 to 75.7)^d1^	26.0 (15.5 to 87.2)
sRAGE (pg/ml)	37.61	<0.001	1,086.0 (237.4 to 5,817.0)^b^	1,113.0 (224.8 to 6,438.0)^a^^,^^c2^	870.9 (157.9 to 2,296.0)	569.0 (249.9 to 1,702.0)	536.9 (323.1 to 1,321.0)	667.6 (297.9 to 1,115.0)

**Notes.**

Data are expressed as median (range).

*χ*^2^ Chi-squared value *P* *P*-value D-CKD diabetic CKD patients ND-CKD non-diabetic CKD patients DM diabetic patients without CKD Non-DM, non-diabetic patients without CKD PGC poor glycaemic control with HbA1c >7% GGC good glycaemic control with HbA1c ≤7% eGFR estimated glomerular filtration rate GPx glutathione peroxidase SOD superoxide dismutase sRAGE soluble RAGE

a *P* < 0.05 versus ND-CKD.

b *P* < 0.001 versus DM (PGC).

c1 *P* < 0.01,

c2 *P* < 0.001 versus DM (GGC).

d1 *P* < 0.05,

d2 *P* < 0.01,

d3 *P* < 0.001 versus Non-DM.

### Renal markers, enzymatic antioxidants and sRAGE in study participants upon stratification of glycaemic control status

The medians of urea, serum creatinine, eGFR, GPx, SOD and sRAGE were compared between groups of study participants in non-parametric Kruskal–Wallis test. Pairwise comparisons using Mann–Whitney *U* test were performed for each parameter that showed overall significance of median difference in Kruskall–Wallis test, except for GPx (Table 2).

D-CKD patients with poor GC, D-CKD patients with good GC and ND-CKD patients showed significantly higher levels of urea and serum creatinine, and lower levels of eGFR than their non-CKD counterparts (*P* < 0.001). No significant differences in these renal markers were observed between study participants of different diabetes status and glycaemic control status in CKD patients, as well as in non-CKD patients.

In general, no significant differences in GPx medians between participant groups were detected. The present study showed no significant differences in plasma SOD activities between D-CKD patients and ND-CKD patients, regardless of their glycaemic control status. The SOD activities were significantly reduced in the CKD patients than in the non-CKD patients. The D-CKD patients with poor GC, D-CKD patients with good GC and ND-CKD patients exhibited significantly lower SOD activities compared to DM patients with poor GC (*P* < 0.001), D-CKD patients with good GC (*P* < 0.001) and non-DM patients (*P* < 0.01) respectively. In this study, SOD was the only parameter that showed statistically significant differences among non-CKD patient groups. The SOD activities in DM patients with good GC was significantly higher than the DM patients with poor GC ( *P* < 0.001) and non-DM patients (*P* < 0.05). On the other hand, the D-CKD patients with good GC exhibited higher plasma sRAGE levels than ND-CKD patients (*P* < 0.05). In addition, sRAGE levels were significantly higher in D-CKD patients with poor GC and good GC than in the DM patients with poor GC (*P* < 0.001) and good GC (*P* < 0.001) respectively.

### Interaction between CKD status and glycaemic control status on GPx, SOD and sRAGE

Table 3 shows the means and standard deviations for GPx, SOD and sRAGE in diabetic subjects. Two-way ANOVA was conducted to analyse the interaction effects of two factors –CKD status and glycaemic control status on GPx, SOD and sRAGE in diabetic subjects (D-CKD and DM patients) (Table 4). Both CKD status and glycaemic control status showed significant interaction effect on SOD (*P* < 0.01), in addition to the separate, significant main effect of each factor. There were no significant interaction effects on GPx and sRAGE. Nonetheless, CKD status showed significant main effect on GPx (*P* < 0.05) and sRAGE (*P* < 0.001) in this group of study participants. The results showed that the means of these parameters were significantly different between CKD patients and non-CKD patients, whereas no significant differences were detected between diabetic subjects with poor GC and good GC. Follow-up tests for SOD such as simple main effect test and interaction comparison were performed following the significant interaction effect.

**Table 3:** Means and standard deviations for GPx, SOD and sRAGE in diabetic subjects.

Parameter	Glycaemic control status	CKD status			
		CKD		Non-CKD	
		Mean	Standard deviation	Mean	Standard deviation
GPx	Poor GC	93.8	27.0	105.4	20.4
	Good GC	96.0	27.8	104.2	26.1
SOD	Poor GC	21.0	12.8	28.3	15.4
	Good GC	20.2	10.6	39.7	15.4
sRAGE	Poor GC	1,326.6	902.3	707.4	386.0
	Good GC	1,657.3	1,407.2	687.0	286.5

**Notes.**

GC glycaemic control GPx glutathione peroxidase SOD superoxide dismutase sRAGE soluble RAGE

**Table 4:** Analysis of interaction between CKD status and glycaemic control status in study participants with diabetes.

Parameters	Factors	*F*	*P*
GPx (nmol/min/ml)	CKD status	4.64	<0.05
	Glycaemic control status	0.01	Not significant
	Interaction between CKD status and glycaemic control status	0.14	Not significant
SOD (U/ml)	CKD status	35.13	<0.001
	Glycaemic control status	5.47	<0.05
	Interaction between CKD status and glycaemic control status	7.22	<0.01
sRAGE (pg/ml)	CKD status	20.41	<0.001
	Glycaemic control status	0.78	Not significant
	Interaction between CKD status and glycaemic control status	1.00	Not significant

**Notes.**

*F* F statistic *P* *P*-value partial *η*^2^ partial eta squared value GPx glutathione peroxidase SOD superoxide dismutase sRAGE soluble RAGE

**Table 5:** Simple main effect test of SOD activities between CKD patients and non-CKD patients for each glycaemic control status.

Glycaemic control status	Mean difference of plasma SOD activities between CKD patients and non-CKD patients	95% confidence interval for the mean difference		Standard error	*P*
		Upper limit	Lower limit		
Poor GC	−7.3	−13.3	−1.4	3.0	0.016^*^
Good GC	−19.5	−26.2	−12.8	3.4	<0.001^*^

**Notes.**

GC glycaemic control SOD superoxide dismutase *P* *P*-value

* The difference is significant at *P*_corr_ <0.025 upon Bonferroni correction.

**Table 6:** Simple main effect test of SOD activities between poor GC and good GC for each CKD status.

CKD status	Mean difference of plasma SOD activities between poor GC and good GC	95% confidence interval for the mean difference		Standard error	*P*
		Upper limit	Lower limit		
CKD	0.88	−4.0	5.5	2.4	0.743
Non-CKD	−11.4	−19.0	−3.8	3.8	0.003^*^

**Notes.**

GC glycaemic control SOD superoxide dismutase *P* *P*-value

* The difference is significant at *P*_corr_ < 0.025 upon Bonferroni correction.

The significance threshold was set at 0.025 for each of the two simple main effects to control for Type I error (Tables 5 and 6). The differences in SOD means were separately examined in simple main effect tests. The SOD activities were significantly lower in CKD patients than in the non-CKD patients for each glycaemic control status (*P*_corr_ < 0.025) (Table 5). However, another simple main effect test (Table 6) showed that the SOD activities were only significantly lower in non-CKD patients with poor GC than in those with good GC (*P*_corr_ < 0.025) whereas such comparison did not show significant difference in CKD patients. These results pointed out that the non-CKD patients with good GC was associated with higher SOD activities, whereas the SOD activities among the CKD patients with poor GC and good GC were comparable.

Interaction comparison was conducted to address whether the absence of CKD and good GC in diabetic subjects are associated with high SOD activities (Table 7). The contrast estimate of 12.2 represents the difference in SOD means between CKD and non-CKD for poor GC minus the difference in SOD means between CKD and non-CKD for good GC (*P* < 0.01). The results of this comparison supported that the non-CKD patients with good GC would benefit from higher SOD activities as compared with other groups of study participants.

**Table 7:** Interaction comparison between CKD and non-CKD for poor GC versus good GC.

Contrast estimate	12.2
95% confidence interval	3.2 to 21.1
*F*	7.22
*P*	<0.01

**Notes.**

GC glycaemic control *F* statistic *P* *P*-value

The results of interaction analysis between CKD status and diabetes status in all of the study participants were presented in Supporting Information files (Tables S1 and S2). The means of GPx, SOD and sRAGE were significantly different between CKD and non-CKD individuals. On the other hand, there were no significant differences in the means of these parameters between diabetic and non-diabetic individuals.

## Discussion

Enzymatic antioxidants and sRAGE have central roles in counteracting oxidative stress and ameliorating pathological features in diabetic nephropathy. The SOD and sRAGE have been shown to alleviate fibrosis and albuminuria as well as improve renal function in rats with diabetic nephropathy (Wendt et al., 2003; Kuo et al., 2015). Maintaining the blood glucose level within the normal range can decrease the risk of albuminuria, which is a risk factor for renal progression (Ruospo et al., 2017). Recent study also showed that higher HbA1c levels were associated with increased cardiovascular mortality in diabetic kidney disease patients (Chang et al., 2016).

In general, the present study showed that activities of enzymatic antioxidants and sRAGE levels were comparable among diabetic CKD patients with poor and good glycaemic control. This suggests that the levels of glycaemic control do not account for the quantitative alterations in GPx, SOD and sRAGE in diabetic CKD patients. Regardless of glycaemic control status, diabetic CKD patients manifested diminished SOD activities and increased sRAGE levels. Thus, the presence of CKD is an important factor that explains such changes of SOD and sRAGE in diabetic patients whereas the different levels of glycaemic control do not affect these parameters in diabetic CKD patients. According to these findings, the presence of CKD may override the effect of glycaemic control in altering the enzymatic antioxidant activities and sRAGE levels.

In this study, CKD patients manifested lower plasma SOD activity than non-CKD patients. This indicates that the patients with renal dysfunction are associated with enhanced oxidative stress. Although this is a speculation, downregulation of SOD expression and enhanced oxidative stress have been reported in murine models with chronic kidney failure that were induced by 5/6 nephrectomy (Vaziri et al., 2003; Ding et al., 2015). The present study showed that SOD activities were higher in patients with well-controlled diabetes. The interaction analysis corroborates the association between increased SOD activities and non-CKD patients with well-controlled diabetes. Therefore, an improvement in glycaemic control in diabetic patients could lead to an increase in SOD activities that helps counteract the increasing oxidative stress in diabetes mellitus or hyperglycaemia. Therefore, an upregulation of SOD could potentially slow down the progression of diabetic CKD in patients with well-controlled diabetes. On the other hand, the presence of CKD negates the effect of glycaemic control on the changes of SOD activities in diabetic CKD patients. This shows that renal dysfunction is associated with lower SOD activities that could not be modulated through controlling hyperglycaemia.

The present study did not show overall differences in GPx activities among the six groups. Based on the results of two-way ANOVA, the diabetic CKD patients manifested lower GPx activities than non-CKD patients, irrespective of their glycaemic control status. This supports the view that GPx activities are decreased in patients with renal dysfunction (Zachara, 2015). Of note, the progression of kidney disease is associated with GPx activity decline (El-Far et al., 2005), indicating the changes in GPx activities depend on the levels of renal function. Although poorly-controlled diabetes is associated with lower GPx activities (Komosińska-Vassev et al., 2005), the comparable GPx activities among CKD patients with poor and good glycaemic control in this study points out that presence of CKD would predispose the diabetic patients to lower activities of enzymatic antioxidants.

Consistent with the current findings, renal failure has predominant contribution over type 2 diabetes towards the augmentation of oxidative stress characterised by the reduction of enzymatic antioxidant activities and the elevation of oxidative damage markers (Kuppusamy et al., 2005). Therefore, the presence of kidney dysfunction is the mainstay of heightened oxidative stress in type 2 diabetic patients. This is evident in type 2 diabetic patients with nephropathy whose enzymatic antioxidants such as GPx, SOD and glutathione reductase were depleted while the levels of malondialdehyde, an oxidative damage marker, were elevated (Kumawat et al., 2013). Our findings also suggest that good glycaemic control does not restore the activities of enzymatic antioxidants in diabetic CKD patients whose antioxidant capacities are commonly diminished. Well-controlled diabetes also does not contribute to an improvement in parameters such as urea, serum creatinine and eGFR in CKD patients. Nevertheless, the risk of albuminuria can be alleviated through tight glycaemic control and antioxidant treatment (Bolignano et al., 2017; Ruospo et al., 2017).

The comparable sRAGE levels in diabetic CKD patients with poor and good glycaemic control in the present study highlights that the presence of CKD negates the impact of glycaemic control on sRAGE levels. These findings concur with a review by Koyama, Yamamoto & Nishizawa (2007) which stated that the sRAGE levels are greatly affected by the presence of CKD in type 2 diabetic patients. Although the current analyses did not show significant interaction effect between CKD and diabetes on sRAGE, it has been postulated that the increased sRAGE levels in diabetes and renal dysfunction may be caused by the increased levels of AGEs (Prasad, 2014). In diabetic milieu, hyperglycaemia results in a concerted increase in RAGE expression and the levels of AGEs (Yao & Brownlee, 2010). The diabetes-induced reactive oxygen species and AGEs induce matrix metalloproteinase-9 which cleaves the cell–surface receptor to form sRAGE, elevating the levels of sRAGE in diabetic patients (Zhang et al., 2008; Prasad, 2014). In addition, the unsatisfactory renal function in the CKD patients may also explain elevated sRAGE levels due to reduced excretion (Kalousová et al., 2006).

The AGEs and sRAGE are mainly excreted by the healthy kidneys (Vlassara, 1994; Kalousová et al., 2007). Under the circumstances of diabetes and kidney disease, the formation of AGEs accelerates and more sRAGE molecules are produced to neutralise the AGEs. However, excess AGEs and sRAGE are unable to be removed due to the declining renal function. In the present study, diabetic CKD patients with poor glycaemic control manifested higher sRAGE levels. This could be attributed to limited excretion and upregulation of sRAGE to thwart the deleterious effects of AGEs. Consistent with this premise, treatment of the diabetic mice with sRAGE reduced the levels of AGEs in vasculature, but did not improve glycaemia in the mice (Park et al., 1998). Chronic hyperglycaemia is associated with increased generation of AGEs and oxidative damage products and exhaustion of antioxidants (Whiting et al., 2008; Yao & Brownlee, 2010). Apparently, well-controlled diabetes can lowers the production of oxidative damage products such as malondialdehyde (De Souza Bastos et al., 2016). These findings supported that sRAGE treatment and good glycaemic control hold promise in ameliorating oxidative stress.

Antidiabetic drugs have been shown to modulate the levels of antioxidants and sRAGE. The treatment with glyburide (sulfonylurea) restored the activities of enzymatic antioxidants such as glutathione-S-transferase and glucose-6-phosphate dehydrogenase in the liver tissues of diabetic rats (Bugdayci et al., 2006). The type 2 diabetic patients who were treated with metformin (biguanide) had lower malondialdehyde levels and higher total antioxidant status (Abdulkadir & Thanoon, 2012). A recent *in vitro* study also demonstrated that metformin could reverse the aldehyde modification-induced inhibition of SOD and upregulate its antioxidant activities in diabetic patients (Lankin et al., 2016). Besides, metformin also prevents oxidative burst through a reduction of reactive oxygen species and the augmentation of enzymatic antioxidant activities in lipopolysaccharide-stimulated human macrophages (Bułdak et al., 2014). The sRAGE levels were raised in type 2 diabetic patients who were treated with a combination of antidiabetic medications such as gliclazide (sulfonylurea), metformin and insulin (Devangelio et al., 2007). Of note, diabetic patients may also use other medications to treat the complications or comorbidities. For instance, diabetic patients who have hypertension and kidney disease may be given perindopril (angiotensin-converting-enzyme inhibitor) that could increase sRAGE levels (Forbes et al., 2005), while the patients with hypercholesterolaemia may be prescribed with statins that could increase the activities of enzymatic antioxidants (Davignon, Jacob & Mason, 2004). Therefore, it is challenging to investigate the modulatory effects of medications on antioxidants and sRAGE due to the vast range of possible combinations and confounding factors.

There are several limitations in the present study. There is a lack of age-matched healthy controls for the comparison of GPx, SOD and sRAGE. In this study, the individuals without CKD and diabetes who were age-matched with the patients were affected by other diseases such as hypertension and cardiovascular diseases. The present study did not show significant differences in these parameters between diabetic patients and non-diabetic patients. In addition, there is a lack of data on AGEs and proteinuria. The levels of AGEs increase as a result of elevated oxidative stress during the course of CKD progression (Stinghen et al., 2016). Besides, proteinuria also deteriorates with CKD progression (Ruggenenti et al., 1998). It is possible that AGEs and proteinuria could be modulated by glycaemic control in diabetic CKD patients. Thus, the impact of glycaemic control on AGEs and proteinuria in diabetic CKD should not be overlooked.

In this study, the glycaemic control levels were set according to the HbA1c targets indicated for general population (Hartz et al., 2006). However, it is recommended to use HbA1c target between 7.5% and 9.0% as the desired glycaemic outcome for the older study participants (Lipska et al., 2016). The reason for imposing less-tight glycaemic control in the older population with diabetes is to minimise the risks and hazards of hypoglycaemic episodes, depression and low body mass index (Panduru et al., 2017).

## Conclusion

Glycaemic control did not account for the changes of the activities or levels of GPx, SOD and sRAGE in diabetic CKD patients. Reduced enzymatic antioxidant capacity and increased sRAGE level are the distinctive characteristics of CKD which are hard to be modulated through good glycaemic control. The presence of CKD would override the effect of glycaemic control on GPx, SOD and sRAGE, whereas the effect of glycaemic control status in determining SOD activities was only pronounced in non-CKD patients. Therefore, reduction of oxidative stress in diabetic CKD patients may be achieved through other means such as antioxidant supplementation and sRAGE therapy.

##  Supplemental Information

10.7717/peerj.4421/supp-1 Supplemental Information 1 Data S1 The dataset contains the information of 214 study participants including the CKD status, diabetes status, glycaemic control status, demographic characteristics, renal function measurement (eGFR), glutathione peroxidase activity, superoxide dismutase activity and soluble RAGE level.

10.7717/peerj.4421/supp-2 Supplemental Information 1 Supplementary information

## Additional Information and Declarations

### Data Availability

The following information was supplied regarding data availability: The raw data has been provided as a Supplemental Dataset.

## References

[ref-1] Abdulkadir AAA, Thanoon IA-J (2012). Comparative effects of glibenclamide and metformin on C-reactive protein and oxidant/antioxidant status in patients with type II diabetes mellitus. Sultan Qaboos University Medical Journal.

[ref-2] Bash LD, Selvin E, Steffes M, Coresh J, Astor BC (2008). Poor glycemic control in diabetes and the risk of incident chronic kidney disease even in the absence of albuminuria and retinopathy: atherosclerosis Risk in Communities (ARIC) Study. Archives of Internal Medicine.

[ref-3] Bolignano D, Cernaro V, Gembillo G, Baggetta R, Buemi M, D’Arrigo G (2017). Antioxidant agents for delaying diabetic kidney disease progression: a systematic review and meta-analysis. PLOS ONE.

[ref-4] Bugdayci G, Altan N, Sancak B, Bukan N, Kosova F (2006). The effect of the sulfonylurea glyburide on glutathione-S-transferase and glucose-6-phosphate dehydrogenase in streptozotocin-induced diabetic rat liver. Acta Diabetologica.

[ref-5] Bułdak Ł, Łabuzek K, Bułdak RJ, Kozłowski M, Machnik G, Liber S, Suchy D, Duława-Bułdak A, Okopień B (2014). Metformin affects macrophages’ phenotype and improves the activity of glutathione peroxidase, superoxide dismutase, catalase and decreases malondialdehyde concentration in a partially AMPK-independent manner in LPS-stimulated human monocytes/macrophages. Pharmacological Reports.

[ref-6] Chang P-Y, Chien L-N, Lin Y-F, Wu M-S, Chiu W-T, Chiou H-Y (2016). Risk factors of gender for renal progression in patients with early chronic kidney disease. Medicine.

[ref-7] Davignon J, Jacob RF, Mason RP (2004). The antioxidant effects of statins. Coronary Artery Disease.

[ref-8] De Souza Bastos A, Graves DT, De Melo Loureiro AP, Júnior CR, Corbi SCT, Frizzera F, Scarel-Caminaga RM, Câmara NO, Andriankaja OM, Hiyane MI, Orrico SRP (2016). Diabetes and increased lipid peroxidation are associated with systemic inflammation even in well-controlled patients. Journal of Diabetes and Its Complications.

[ref-9] Devangelio E, Santilli F, Formoso G, Ferroni P, Bucciarelli L, Michetti N, Clissa C, Ciabattoni G, Consoli A, Davì G (2007). Soluble RAGE in type 2 diabetes: association with oxidative stress. Free Radical Biology & Medicine.

[ref-10] Ding W, Wang B, Zhang M, Gu Y (2015). Tempol, a superoxide dismutase-mimetic drug, ameliorates progression of renal disease in CKD mice. Cellular Physiology and Biochemistry.

[ref-11] El-Far MA, Bakr MA, Farahat SE, Abd El-Fattah EA (2005). Glutathione peroxidase activity in patients with renal disorders. Clinical and Experimental Nephrology.

[ref-12] Forbes JM, Thorpe SR, Thallas-Bonke V, Pete J, Thomas MC, Deemer ER, Bassal S, El-Osta A, Long DM, Panagiotopoulos S, Jerums G, Osicka TM, Cooper ME (2005). Modulation of soluble receptor for advanced glycation end products by angiotensin-converting enzyme-1 inhibition in diabetic nephropathy. Journal of the American Society of Nephrology.

[ref-13] Fukai T, Ushio-Fukai M (2011). Superoxide dismutases: role in redox signaling, vascular function, and diseases. Antioxidants & Redox Signaling.

[ref-14] Gkogkolou P, Böhm M (2012). Advanced glycation end products: key players in skin aging?. Dermato-Endocrinology.

[ref-15] Hartz A, Kent S, James P, Xu Y, Kelly M, Daly J (2006). Factors that influence improvement for patients with poorly controlled type 2 diabetes. Diabetes Research and Clinical Practice.

[ref-16] Kalea AZ, Schmidt AM, Hudson BI (2009). RAGE: a novel biological and genetic marker for vascular disease. Clinical Science.

[ref-17] Kalousová M, Bartosová K, Zima T, Skibová J, Teplan V, Viklický O (2007). Pregnancy-associated plasma protein a and soluble receptor for advanced glycation end products after kidney transplantation. Kidney & Blood Pressure Research.

[ref-18] Kalousová M, Hodková M, Kazderová M, Fialová J, Tesar V, Dusilová-Sulková S, Zima T (2006). Soluble receptor for advanced glycation end products in patients with decreased renal function. American Journal of Kidney Diseases: the Official Journal of the National Kidney Foundation.

[ref-19] Komosińska-Vassev K, Olczyk K, Olczyk P, Winsz-Szczotka K (2005). Effects of metabolic control and vascular complications on indices of oxidative stress in type 2 diabetic patients. Diabetes Research and Clinical Practice.

[ref-20] Koyama H, Yamamoto H, Nishizawa Y (2007). RAGE and soluble RAGE: potential therapeutic targets for cardiovascular diseases. Molecular Medicine.

[ref-21] Kumawat M, Sharma TK, Singh I, Singh N, Ghalaut VS, Vardey SK, Shankar V (2013). Antioxidant enzymes and lipid peroxidation in type 2 diabetes mellitus patients with and without nephropathy. North American Journal of Medical Sciences.

[ref-22] Kuo C-W, Shen C-J, Tung Y-T, Chen H-L, Chen Y-H, Chang W-H, Cheng K-C, Yang S-H, Chen C-M (2015). Extracellular superoxide dismutase ameliorates streptozotocin-induced rat diabetic nephropathy via inhibiting the ROS/ERK1/2 signaling. Life Sciences.

[ref-23] Kuppusamy UR, Indran M, Ahmad T, Wong SW, Tan SY, Mahmood AA (2005). Comparison of oxidative damage in Malaysian end-stage renal disease patients with or without non-insulin-dependent diabetes mellitus. Clinica Chimica Acta.

[ref-24] Lankin VZ, Konovalova GG, Tikhaze AK, Shumaev KB, Belova Kumskova EM, Grechnikova MA, Viigimaa M (2016). Aldehyde inhibition of antioxidant enzymes in the blood of diabetic patients. Journal of Diabetes.

[ref-25] Levey AS, Coresh J, Balk E, Kausz AT, Levin A, Steffes MW, Hogg RJ, Perrone RD, Lau J, Eknoyan G (2003). National Kidney Foundation practice guidelines for chronic kidney disease: evaluation, classification, and stratification. Annals of Internal Medicine.

[ref-26] Levey AS, Coresh J, Greene T, Stevens LA, Zhang YL, Hendriksen S, Kusek JW, Van Lente F (2006). Using standardized serum creatinine values in the modification of diet in renal disease study equation for estimating glomerular filtration rate. Annals of Internal Medicine.

[ref-27] Lipska KJ, Krumholz H, Soones T, Lee SJ (2016). Polypharmacy in the aging patient: a review of glycemic control in older adults with type 2 diabetes. JAMA.

[ref-28] Panduru NM, Nistor I, Groop P-H, Van Biesen W, Farrington K, Covic A (2017). Considerations on glycaemic control in older and/or frail individuals with diabetes and advanced kidney disease. Nephrology Dialysis Transplantation.

[ref-29] Park L, Raman KG, Lee KJ, Lu Y, Ferran LJ, Chow WS, Stern D, Schmidt AM (1998). Suppression of accelerated diabetic atherosclerosis by the soluble receptor for advanced glycation endproducts. Nature Medicine.

[ref-30] Prasad K (2014). Low levels of serum soluble receptors for advanced glycation end products, biomarkers for disease state: myth or reality. The International Journal of Angiology.

[ref-31] Ramasamy R, Yan SF, Schmidt AM (2009). RAGE: therapeutic target and biomarker of the inflammatory response–the evidence mounts. Journal of Leukocyte Biology.

[ref-32] Ruggenenti P, Perna A, Mosconi L, Pisoni R, Remuzzi G (1998). Urinary protein excretion rate is the best independent predictor of ESRF in non-diabetic proteinuric chronic nephropathies. Kidney International.

[ref-33] Ruospo M, Saglimbene VM, Palmer SC, De Cosmo S, Pacilli A, Lamacchia O, Cignarelli M, Fioretto P, Vecchio M, Craig JC, Strippoli GFM (2017). Glucose targets for preventing diabetic kidney disease and its progression. The Cochrane Database of Systematic Reviews.

[ref-34] Stinghen AEM, Massy ZA, Vlassara H, Striker GE, Boullier A (2016). Uremic toxicity of advanced glycation end products in CKD. Journal of the American Society of Nephrology.

[ref-35] The International Expert Committee (2009). International expert committee report on the role of the A1C assay in the diagnosis of diabetes. Diabetes Care.

[ref-36] Vaziri ND, Dicus M, Ho ND, Boroujerdi-Rad L, Sindhu RK (2003). Oxidative stress and dysregulation of superoxide dismutase and NADPH oxidase in renal insufficiency. Kidney International.

[ref-37] Vlassara H (1994). Serum advanced glycosylation end products: a new class of uremic toxins?. Blood Purification.

[ref-38] Wendt TM, Tanji N, Guo J, Kislinger TR, Qu W, Lu Y, Bucciarelli LG, Rong LL, Moser B, Markowitz GS, Stein G, Bierhaus A, Liliensiek B, Arnold B, Nawroth PP, Stern DM, D’Agati VD, Schmidt AM (2003). RAGE drives the development of glomerulosclerosis and implicates podocyte activation in the pathogenesis of diabetic nephropathy. The American Journal of Pathology.

[ref-39] Whiting PH, Kalansooriya A, Holbrook I, Haddad F, Jennings PE (2008). The relationship between chronic glycaemic control and oxidative stress in type 2 diabetes mellitus. British Journal of Biomedical Science.

[ref-40] Wong FN, Tan JAMA, Keng TC, Ng KP, Chua KH, Kuppusamy UR (2016b). Association between plasma soluble RAGE and renal function is unaffected by medication usage and enzymatic antioxidants in chronic kidney disease with type 2 diabetes. Clinica Chimica Acta.

[ref-41] Wong MG, Perkovic V, Chalmers J, Woodward M, Li Q, Cooper ME, Hamet P, Harrap S, Heller S, MacMahon S, Mancia G, Marre M, Matthews D, Neal B, Poulter N, Rodgers A, Williams B, Zoungas S, ADVANCE-ON Collaborative Group (2016a). Long-term benefits of intensive glucose control for preventing end-stage kidney disease: ADVANCE-ON. Diabetes Care.

[ref-42] Yao D, Brownlee M (2010). Hyperglycemia-induced reactive oxygen species increase expression of the receptor for advanced glycation end products (RAGE) and RAGE ligands. Diabetes.

[ref-43] Zachara BA (2015). Selenium and selenium-dependent antioxidants in chronic kidney disease. Advances in Clinical Chemistry.

[ref-44] Zhang L, Bukulin M, Kojro E, Roth A, Metz VV, Fahrenholz F, Nawroth PP, Bierhaus A, Postina R (2008). Receptor for advanced glycation end products is subjected to protein ectodomain shedding by metalloproteinases. The Journal of Biological Chemistry.

