# Supplementary material for: Glycaemic control in type 2 diabetic patients with chronic kidney disease: the impacts on enzymatic antioxidants and soluble RAGE
Source: PeerJ. 2018 Mar 30;6:e4421. doi: 10.7717/peerj.4421 (PMC5880175; doi:10.7717/peerj.4421)
Supplement: Supplemental Information 1 [file peerj-06-4421-s002.docx]

**Supplementary Information**

**Table S1.** Means and standard deviations for GPx, SOD and sRAGE in study participants

| **Parameter** | **Diabetes status** | **CKD status** | | | |
| --- | --- | --- | --- | --- | --- |
|  |  | **CKD** | | **Non-CKD** | |
|  |  | **Mean** | **Standard deviation** | **Mean** | **Standard deviation** |
| GPx | Diabetic | 94.8 | 27.2 | 104.9 | 22.8 |
|  | Non-diabetic | 99.5 | 30.5 | 109.8 | 21.1 |
| SOD | Diabetic | 20.6 | 11.8 | 33.3 | 16.3 |
|  | Non-diabetic | 20.1 | 9.4 | 32.7 | 19.0 |
| sRAGE | Diabetic | 1477.7 | 1166.9 | 698.5 | 342.9 |
|  | Non-diabetic | 1060.4 | 605.1 | 706.8 | 258.8 |

In the table, GPx, glutathione peroxidase, SOD, superoxide dismutase; sRAGE, soluble RAGE.

**Table S2.** Analysis of interaction between CKD status and glycaemic control status in study participants

| **Parameters** | **Factors** | **F** | ***P*** |
| --- | --- | --- | --- |
| GPx (nmol/min/ml) | CKD status | 5.11 | < 0.05 |
|  | Diabetes status | 1.15 | Not significant |
|  | Interaction between CKD status and diabetes control status | 0.00 | Not significant |
| SOD (U/ml) | CKD status | 31.50 | < 0.001 |
|  | Diabetes status | 0.07 | Not significant |
|  | Interaction between CKD status and diabetes control status | 0.00 | Not significant |
| sRAGE (pg/ml) | CKD status | 13.34 | < 0.001 |
|  | Diabetes status | 1.74 | Not significant |
|  | Interaction between CKD status and diabetes control status | 1.88 | Not significant |

In the table, F, F statistic; *P*, P-value; partial η^2^, partial eta squared value; GPx, glutathione peroxidase; SOD, superoxide dismutase; sRAGE, soluble RAGE.
